# *PRAME* expression and clinical outcome of breast cancer

**DOI:** 10.1038/sj.bjc.6604494

**Published:** 2008-07-22

**Authors:** M T Epping, A A M Hart, A M Glas, O Krijgsman, R Bernards

**Affiliations:** 1 Division of Molecular Carcinogenesis and Centre for Biomedical Genetics, The Netherlands Cancer Institute, Plesmanlaan 121, Amsterdam 1066 CX, The Netherlands; 2 Department of Radiation Oncology, The Netherlands Cancer Institute, Plesmanlaan 121, Amsterdam 1066 CX, The Netherlands; 3 Agendia BV, Louwesweg 6 9D, Amsterdam 1066 EC, The Netherlands

**Keywords:** PRAME, tumour antigen, prognosis, metastasis, survival, microarray

## Abstract

The tumour antigen PReferentially expressed Antigen of MElanoma (PRAME) is expressed in a variety of malignancies, including breast cancer. We have analysed *PRAME* gene expression in relation to clinical outcome for 295 primary breast cancer patients. Kaplan–Meier survival curves show a correlation of *PRAME* expression levels with increased rates of distant metastases and decreased overall patient survival. This correlation existed both for the entire patient group (*n*=295) and for the subgroup of patients (*n*=185) who did not receive adjuvant chemotherapy. Multivariable analysis indicated that *PRAME* is an independent marker of shortened metastasis-free interval in patients who did not receive adjuvant chemotherapy. *PRAME* expression was associated with tumour grade and negative oestrogen receptor status. We conclude that *PRAME* expression is a prognostic marker for clinical outcome of breast cancer, independent of traditional clinicopathological markers.

The expression of preferentially expressed antigen of melanoma (PRAME) has been detected in a variety of cancers including breast cancer, but its expression is low or absent in normal tissues (Ikeda *et al*, 1997). The protein PRAME was first detected as a tumour antigen in cells isolated from a melanoma, and high *PRAME* expression has been detected in 88–95% of primary melanomas (Ikeda *et al*, 1997). PRAME can inhibit retinoic acid (RA) signalling leading to resistance of melanoma cells to proliferation arrest induced by RA (Epping *et al*, 2005). The function of PRAME in breast cancer and other cancers in which it is expressed is still elusive (Epping and Bernards, 2006).

Among the tumour types expressing *PRAME* are breast cancers, lung cancers, sarcomas, Wilms' tumours, renal carcinomas, medulloblastomas, head-and-neck cancers, lymphomas, and several types of leukaemias (Ikeda *et al*, 1997; Neumann *et al*, 1998; Li *et al*, 2002; Steinbach *et al*, 2002b; Boon *et al*, 2003; Radich *et al*, 2006; Willenbrock *et al*, 2006). Although many reports have focused on the detection of *PRAME* mRNA transcripts, there are few studies that have linked gene expression data directly to clinical information. The expression of *PRAME* is associated with poor prognosis in neuroblastoma: high *PRAME* expression is associated with more advanced tumour stage, higher ages of patients at diagnosis, and poor clinical outcome (Oberthuer *et al*, 2004). *PRAME* expression has been linked to good prognosis in paediatric AML (Steinbach *et al*, 2002a), but other studies did not find a significant correlation (Paydas *et al*, 2005).

We previously identified a gene expression profile that is associated with the risk of early distant metastases in young breast cancer patients (van ‘t Veer *et al*, 2002). The tumours of 78 women with sporadic lymph node-negative breast cancer were selected to search for a prognostic signature in their gene expression profiles. We found that 231 genes were significantly associated with disease outcome, one of which was *PRAME*. Serial computational analyses of the data were conducted to generate a ‘prognosis classifier’ comprising an optimised number of 70 marker genes. *PRAME* was part of the set of 231 ‘significant prognosis reporter genes’, but not of the optimal set of 70, which together constitute the prognosis classifier (van ‘t Veer *et al*, 2002). This classifier allowed the categorisation of patients in a ‘good-prognosis’ and a ‘poor-prognosis’ group, as defined by the occurrence of distant metastases within 5 years after initial diagnosis (van ‘t Veer *et al*, 2002).

In a subsequent validation study, the tumours of a series of 295 consecutive women with breast cancer were used to validate the 70-gene prognostic profile (van de Vijver *et al*, 2002). The genome-wide gene expression profiles of these tumours demonstrated the prognostic power of this profile in predicting the outcome of disease. The poor-prognosis signature was the strongest predictor of the likelihood of distant metastases in all patients, with a more accurate prediction of disease outcome than clinicopathological criteria and the NIH and St Gallen criteria (van de Vijver *et al*, 2002). The prognosis classifier could be used to select effectively those high-risk patients who would benefit from adjuvant therapy, while reducing the number of patients who receive unnecessary treatment and may suffer from the side effects. Thus, the prognostic profile potentially provides a powerful tool to tailor adjuvant systemic treatment of breast cancer (Buyse *et al*, 2006). Moreover, the prognostic power of the 70-gene profile indicates that the ability to metastasise to distant sites is an early and inherent genetic property of breast cancer, which argues against the widely accepted idea that metastatic potential is acquired relatively late during multistep tumorigenesis (Bernards and Weinberg, 2002).

Because *PRAME* was not part of the 70-gene prognosis classifier, we have analysed the gene expression data set of the 295 breast cancer patients from our previous study (van de Vijver *et al*, 2002) for the expression levels of *PRAME* and found a remarkable association between *PRAME* expression and poor clinical outcome. We discuss these findings in the context of the recent insights in *PRAME* function.

## Materials and Methods

### Selection of patients

Tumours from a series of 295 consecutive women with breast cancer were selected from the fresh-frozen tissue bank of the Netherlands Cancer Institute according to the following criteria as described previously (van de Vijver *et al*, 2002). The tumour was primary invasive breast carcinoma less than 5 cm in diameter at pathological examination (pT1 or pT2); the apical axillary lymph nodes were tumour-negative, as determined by a biopsy of the infraclavicular lymph nodes; the age at diagnosis was 52 years or younger; the calendar year of diagnosis was between 1984 and 1995; and there was no previous history of cancer, except nonmelanoma skin cancer. All patients had been treated by modified radical mastectomy or breast-conserving surgery, including dissection of the axillary lymph nodes, followed by radiotherapy if indicated. Among the 295 patients, 151 had lymph node-negative disease (results on pathological examination, pN0) and 144 had lymph node-positive disease (pN+). All patients were assessed at least annually for a period of at least 5 years. Follow-up information was extracted from the medical registry of the Netherlands Cancer Institute. The median follow-up among all 295 patients was 6.7 years (range, 0.05–18.3). There were no missing data. The study was approved by the medical ethics committee of the Netherlands Cancer Institute.

### Isolation of RNA and microarray expression profiling

The isolation of RNA, labelling of complementary RNA (cRNA), hybridisation of labelled cRNA to 25 000 gene arrays, and assessment of expression ratios were all performed as previously described (Hughes *et al*, 2001; van ‘t Veer *et al*, 2002). In brief, tumour material was snap-frozen in liquid nitrogen within 1 h after surgery. Frozen sections were stained with haematoxylin and eosin; only samples that had more than 50 per cent tumour cells were selected. Thirty 30-*μ*m sections were used for the isolation of RNA. Total RNA was isolated with RNAzolB and dissolved in RNase-free water. Then 25 *μ*g of total RNA was treated with DNase with use of the Qiagen RNase-free DNase kit and RNeasy spin columns, and the RNA was then dissolved in RNase-free water to a final concentration of 0.2 *μ*g per microlitre. Complementary RNA was generated by *in vitro* transcription with the use of T7 RNA polymerase and 5 *μ*g of total RNA and labelled with Cy3 or Cy5 (Cy Dye; Amersham Pharmacia Biotech, Piscataway, NJ, USA). Five micrograms of Cy-labelled cRNA from one breast cancer tumour was mixed with the same amount of reverse-colour Cy-labelled product from a reference pool that consisted of an equal amount of cRNA from each patient. Labelled cRNAs were fragmented to an average size of approximately 50–100 nucleotides by heating the samples to 60°C in the presence of 10 mM zinc chloride and adding a hybridisation buffer containing 1 M sodium chloride, 0.5 per cent sodium sarcosine, 50 mM morpholino-ethane sulphonic acid (pH 6.5), and formamide (final concentration, 30 per cent at 40°C); the final volume was 3 ml. The microarrays included 24 479 biologic oligonucleotides as well as 1281 control probes. The hybridisations were performed in duplicates and with colour reversal. After hybridisation, the slides were washed and scanned with a confocal laser scanner (Agilent Technologies, Palo Alto, CA, USA). Fluorescence intensities on scanned images were quantified, and the values were corrected for the background level and normalised.

### Statistical methods

**Figure 1:**
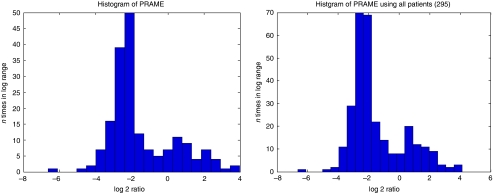
Histograms of *PRAME* expression levels (log 2 ratios) for patients without adjuvant treatment (left; *n*=185) and for all the patients (right; *n*=295).

The *PRAME* expression data were linked to the clinical database on the basis of the Rosetta Bioinformatics patient identification number (rosid). The probe sequence for *PRAME* was checked using BLAST and was found to code only for *PRAME* and did not match any other sequence. *PRAME* expression was quantified as the logarithm of the intensity ratio with respect to a standard pool of breast cancers. A normal probability plot indicated the existence of two subgroups of patients with either relatively high or low *PRAME* expression. An expectation–maximisation (EM) algorithm was used to estimate the mean values and standard deviations (s.d.) of the log 2 ratio subgroups, which were treated as normal distributions, an assumption that was supported by histograms (Figure 1). The distance of every sample from the mean of both groups measured in s.d. of that group was calculated. The samples were assigned to the group with the smallest distance in s.d. of that group. This method assigned 98 samples (33%) to the high-expression group and 197 samples (67%) to the low-expression group. The cutoff value for *PRAME* expression levels in log 2 was −1.45, which equals −0.4365 in log 10.

Time in years to distant metastasis as first event was measured from the date of diagnosis of the primary tumour. For metastasis, the occurrence of a metastasis before any other failure (locoregional recurrence or contralateral breast cancer or death) was counted as an event, whereas all other patients were censored at the time of another type of failure or end of follow-up. For overall survival, death from any cause was counted as an event, whereas patients still alive at the end of follow-up were censored at that time. The *P*-values shown for the Kaplan–Meier (KM) curves were calculated using a log rank test. Cox regression analysis was used to analyse the prognostic value of *PRAME* in addition to that of known clinicopathological prognosticators.

## Results

The expression levels of *PRAME* mRNA in the primary breast cancer biopsies of 295 patients used in our previous study (van de Vijver *et al*, 2002) were analysed and matched to the clinical follow-up data. There were 110 patients who received adjuvant systemic chemotherapy, the majority of whom had lymph node-positive disease (van de Vijver *et al*, 2002). These patients were separated from the whole group, leaving 185 patients who had not received adjuvant chemotherapy. In both cohorts of patients, two subgroups for *PRAME* expression existed, with relatively low and relatively high *PRAME* levels. Histograms were made to confirm the existence of two subgroups for *PRAME* expression in each cohort (Figure 1). Ninety-eight breast cancer samples (33%) were assigned to the high-expression group and 197 samples (67%) to the low-expression group, using a cutoff value of −1.45 in log 2, which equals −0.4365 in log 10 (see Materials and methods).

**Figure 2:**
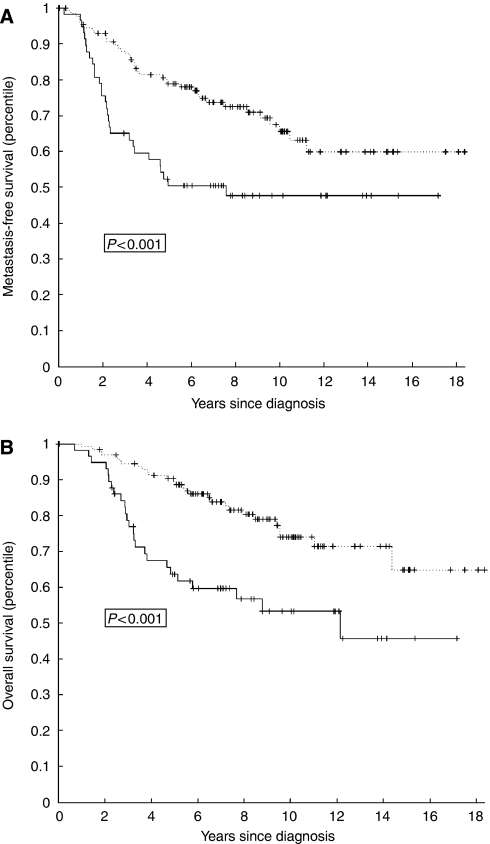
Kaplan–Meier plots for metastasis-free interval (**A**) and overall survival (**B**) for patients who did not receive adjuvant chemotherapy (*n*=185) categorised by *PRAME* mRNA levels (high PRAME: solid line; low PRAME: dashed line). *P*-values were calculated by using a log rank test.

To evaluate the clinical relevance of *PRAME* expression, Kaplan–Meier plots for overall survival and metastasis-free interval were made. For the group of patients who did not receive adjuvant chemotherapy, there were significant associations between *PRAME* expression levels and shortened overall survival (*P*<0.001), and *PRAME* expression levels and shortened metastasis-free interval (*P*<0.001) (Figure 2). These data indicate that *PRAME* expression is a prognostic marker for breast cancer progression.

**Figure 3:**
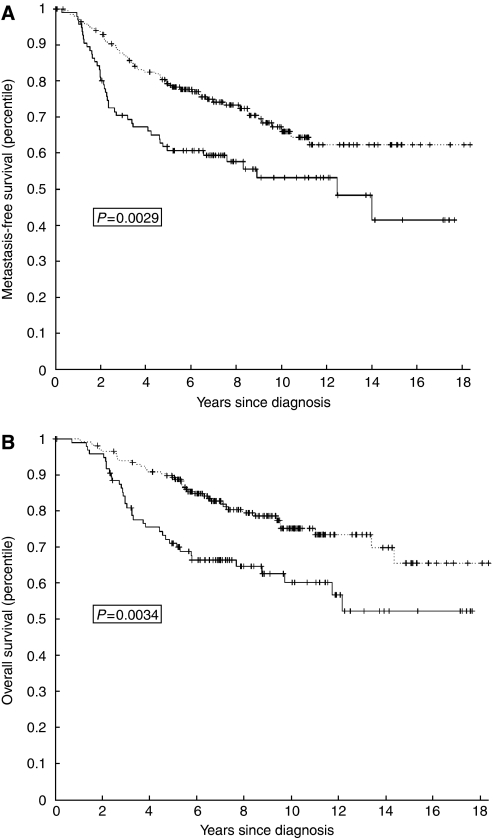
Kaplan–Meier plots for metastasis-free interval (**A**) and overall survival (**B**) for all patients (*n*=295) categorised by *PRAME* mRNA levels (high PRAME: solid line; low PRAME: dashed line). *P*-values were calculated by using a log rank test.

Subsequently, we evaluated whether there was a relation between *PRAME* expression levels and clinical follow-up in the whole group of 295 patients. There were negative associations between *PRAME* expression levels and overall survival (*P*=0.0034) and metastasis-free interval (*P*=0.0029), that is high *PRAME* levels were associated with poor outcome (Figure 3). Thus, *PRAME* mRNA expression levels were inversely correlated with survival in all the patients, irrespective of treatment, and KM plots and *P*-values showed a more significant separation between high- and low-risk samples for the patient group without adjuvant chemotherapy compared to all 295 patients.

**Figure 4:**
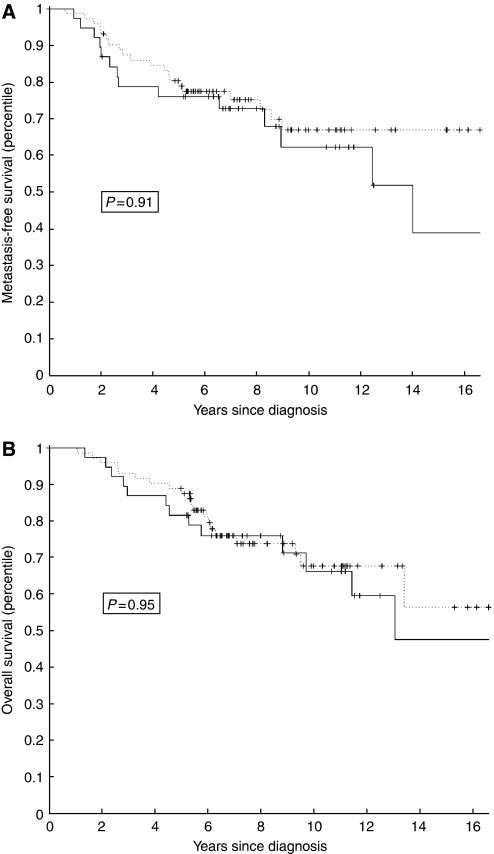
Kaplan–Meier plots for metastasis-free interval (**A**) and overall survival (**B**) for patients who received adjuvant chemotherapy (*n*=110) categorised by *PRAME* mRNA levels (high PRAME: solid line; low PRAME: dashed line). *P*-values were calculated by using a log rank test.

To determine whether *PRAME* expression is predictive for adjuvant chemotherapy response, the subgroup of 110 patients who received chemotherapy was analysed for *PRAME* expression and clinical outcome. Remarkably, there was no significant difference between the treated patients with high and low *PRAME* expressions with regard to overall survival (*P*=0.95) and metastasis-free interval (*P*=0.91) (Figure 4). These data indicate that the patients with high *PRAME* expression have had significant benefit from the adjuvant chemotherapy and that in this patient cohort, *PRAME* expression was predictive for response to adjuvant chemotherapy.

Expression of *PRAME* was compared with clinicopathological characteristics in a multivariable analysis. Independent prognostic factors for metastasis-free interval were *PRAME* expression (*P*=0.006), age, lymph node status, mastectomy and tumour grade, and vascularisation for the 185 patients who did not receive chemotherapy (Table 1). In all, for the 295 patients, age, lymph node status, tumour size, grade and vascularisation, and chemotherapy were independent prognostic factors for metastasis-free interval (Table 2). *PRAME* expression was not significant in this group (*P*=0.11), probably due to the strong effect of chemotherapy.

To search for a possible relation between *PRAME* expression and clinicopathological tumour characteristics, associations between *PRAME* expression and clinical variables (age, tumour diameter, number of positive axillary nodes, histologic grade, oestrogen receptor (ER) status, vascular invasion, lymphatic infiltration) were analysed. Only for grade and ER status, evidence for an association with *PRAME* was found. In the 295-breast cancer patient group, high *PRAME* expression levels were associated with poorly differentiated tumours and low *PRAME* expression with well-differentiated tumours (Kruskal–Wallis test: *P*=0.0005). Furthermore, the expression of *PRAME* was associated with negative ER status, as ER-negative patients had mostly high *PRAME* expression, whereas ER-positive patients had mostly low *PRAME* expression (Mann–Whitney test: *P*<0.0001). Although significant, this negative association of *PRAME* with ER was not consistent among all patients, which may be explained by the two patient subgroups with respect to *PRAME* mRNA expression (Figure 1). The lymph node status of patients was not directly associated with *PRAME* expression (*P*=0.678 for the nontreated patients; *P*=0.691 for all patients).

In conclusion, our analyses provide evidence for an association of high *PRAME* expression levels with poor clinical outcome of premenopausal breast cancer with increased rates of distant metastases and lower rates of overall survival.

## Discussion

In the present study, we have evaluated the prognostic value of *PRAME* mRNA expression in 295 primary breast cancer biopsies. The full-genome gene expression data of these patients were previously used to validate the ‘poor-prognosis profile’ of 70 genes (van de Vijver *et al*, 2002; van ‘t Veer *et al*, 2002). Using this large data set, we have demonstrated that *PRAME* is a prognostic marker for metastasis-free interval and overall survival. These data are reminiscent of a report showing that the expression of *PRAME* is associated with poor prognosis in neuroblastoma (Oberthuer *et al*, 2004).

Recently, an association between *PRAME* expression and unfavourable disease outcome was shown in a study involving 103 breast cancer biopsies in which *PRAME* mRNA was detected in ∼53% of tumour specimens (Doolan *et al*, 2008). The presence of *PRAME* expression was associated with shortened disease-free survival and overall survival in all breast cancer cases (Doolan *et al*, 2008). In the cases in which adjuvant chemotherapy was administrated, an association existed between *PRAME* expression and shortened relapse-free survival (Doolan *et al*, 2008). In our study, we provide evidence that *PRAME* expression is a prognostic marker for metastasis-free interval and overall survival in primary breast cancer. We also demonstrate that the strongest correlation exists for patients who did not receive adjuvant chemotherapy, indicating that *PRAME* has prognostic power in primary breast cancer. Therefore, the data of the previous study (Doolan *et al*, 2008) are fully consistent with the conclusion of the present larger study based on 295 patients and a subgroup of 185 patients who did not receive chemotherapy. Our data differ from those of Doolan *et al* (2008), in that we find that *PRAME* expression predicts benefit of chemotherapy (Figure 4). However, given this discrepancy, additional tumour series should be evaluated to address the significance of *PRAME* as a biomarker of chemotherapy response.

We have found recently that PRAME is a corepressor of the RA receptor and harbours seven ‘nuclear receptor boxes’, motifs that allow interaction with nuclear receptors (Epping *et al*, 2005). This finding begs the question whether the function of PRAME in breast cancer progression is also to inhibit the function of specific nuclear receptors. In our study, *PRAME* expression was found to be higher in the ER-negative breast tumours, suggesting that PRAME does not act on ER. Consistent with this, we did not observe an effect of *PRAME* expression on ER or progesterone receptor activity (Epping *et al*, 2005). Similarly, we observed lower RAR*α* levels in tumours having high *PRAME* expression, suggesting that PRAME may also not act through RAR*α* in breast cancer. Which nuclear receptor (if any) is targeted by PRAME in breast cancer remains elusive at present.

## Figures and Tables

**Table 1:** Multivariable proportional-hazard analysis of the risk of distant metastases as a first event in patients who did not receive chemotherapy (*n*=185)

**Variable**	**Hazard ratio (95% CI^a^)**	***P*-value**
Age (per 10-year increment)	0.58 (0.39–0.87)	0.008
Lymph node status (per positive node)	1.16 (1.02–1.30)	0.02
Tumour diameter (per 10 cm)	1.15 (0.82–1.61)	0.42
		
*Tumour grade*		
Grade 2 (*vs* grade 1)	2.76 (1.09–6.99)	0.03
Grade 3 (*vs* grade 1)	2.80 (1.07–7.33)	0.04
		
*Vascular invasion*		
1–3 vessels (*vs* 0 vessels)	0.45 (0.11–1.90)	0.28
>3 vessels (*vs* 0 vessels)	2.14 (1.25–3.67)	0.006
Oestrogen receptor expression	1.02 (0.61–1.70)	0.95
Mastectomy (*vs* breast-conserving therapy)	1.92 (1.08–3.40)	0.03
Hormonal treatment (*vs* no hormonal treatment)	1.05 (0.40–2.79)	0.92
*PRAME* high expression (*vs* low expression)	2.26 (1.27–4.01)	0.006

a CI denotes confidence interval.

**Table 2:** Multivariable proportional-hazard analysis of the risk of distant metastases as a first event in all patients (*n*=295)

**Variable**	**Hazard ratio (95% CI^a^)**	***P*-value**
Age (per 10-year increment)	0.71 (0.51–0.99)	0.04
Lymph node status (per positive node)	1.12 (1.03–1.22)	0.01
Tumour diameter (per 10 cm)	1.32 (1.03–1.68)	0.03
		
*Tumour grade*		
Grade 2 (*vs* grade 1)	2.27 (1.06–4.85)	0.04
Grade 3 (*vs* grade 1)	2.36 (1.09–5.11)	0.03
		
*Vascular invasion*		
1–3 vessels (*vs* 0 vessels)	0.81 (0.34–1.94)	0.64
>3 vessels (*vs* 0 vessels)	1.66 (1.03–2.66)	0.04
Oestrogen receptor expression	0.76 (0.50–1.16)	0.21
Mastectomy (*vs* breast-conserving therapy)	1.28 (0.80–2.04)	0.30
Chemotherapy (*vs* no chemotherapy)	0.39 (0.22–0.68)	0.001
Hormonal treatment (*vs* no hormonal treatment)	0.74 (0.33–1.66)	0.47
*PRAME* high expression (*vs* low expression)	1.47 (0.92–2.37)	0.11

a CI denotes confidence interval.

## References

[bib1] Bernards R, Weinberg RA (2002) A progression puzzle. Nature 418: 823 12192390 10.1038/418823a

[bib2] Boon K, Edwards JB, Siu IM, Olschner D, Eberhart CG, Marra MA, Strausberg RL, Riggins GJ (2003) Comparison of medulloblastoma and normal neural transcriptomes identifies a restricted set of activated genes. Oncogene 22: 7687–7694 14576832 10.1038/sj.onc.1207043

[bib3] Buyse M, Loi S, van't Veer L, Viale G, Delorenzi M, Glas AM, d'Assignies MS, Bergh J, Lidereau R, Ellis P, Harris A, Bogaerts J, Therasse P, Floore A, Amakrane M, Piette F, Rutgers E, Sotiriou C, Cardoso F, Piccart MJ (2006) Validation and clinical utility of a 70-gene prognostic signature for women with node-negative breast cancer. J Natl Cancer Inst 98: 1183–1192 16954471 10.1093/jnci/djj329

[bib4] Doolan P, Clynes M, Kennedy S, Mehta JP, Crown J, O'Driscoll L (2008) Prevalence and prognostic and predictive relevance of PRAME in breast cancer. Breast Cancer Res Treat 109: 359–365 17624586 10.1007/s10549-007-9643-3

[bib5] Epping MT, Bernards R (2006) A causal role for the human tumor antigen preferentially expressed antigen of melanoma in cancer. Cancer Res 66: 10639–10642 17108098 10.1158/0008-5472.CAN-06-2522

[bib6] Epping MT, Wang L, Edel MJ, Carlee L, Hernandez M, Bernards R (2005) The human tumor antigen PRAME is a dominant repressor of retinoic acid receptor signaling. Cell 122: 835–847 16179254 10.1016/j.cell.2005.07.003

[bib7] Hughes TR, Mao M, Jones AR, Burchard J, Marton MJ, Shannon KW, Lefkowitz SM, Ziman M, Schelter JM, Meyer MR, Kobayashi S, Davis C, Dai H, He YD, Stephaniants SB, Cavet G, Walker WL, West A, Coffey E, Shoemaker DD, Stoughton R, Blanchard AP, Friend SH, Linsley PS (2001) Expression profiling using microarrays fabricated by an ink-jet oligonucleotide synthesizer. Nat Biotechnol 19: 342–347 11283592 10.1038/86730

[bib8] Ikeda H, Lethe B, Lehmann F, van Baren N, Baurain JF, de Smet C, Chambost H, Vitale M, Moretta A, Boon T, Coulie PG (1997) Characterization of an antigen that is recognized on a melanoma showing partial HLA loss by CTL expressing an NK inhibitory receptor. Immunity 6: 199–208 9047241 10.1016/s1074-7613(00)80426-4

[bib9] Li CM, Guo M, Borczuk A, Powell CA, Wei M, Thaker HM, Friedman R, Klein U, Tycko B (2002) Gene expression in Wilms' tumor mimics the earliest committed stage in the metanephric mesenchymal–epithelial transition. Am J Pathol 160: 2181–2190 12057921 10.1016/S0002-9440(10)61166-2 PMC1850829

[bib10] Neumann E, Engelsberg A, Decker J, Storkel S, Jaeger E, Huber C, Seliger B (1998) Heterogeneous expression of the tumor-associated antigens RAGE-1, PRAME, and glycoprotein 75 in human renal cell carcinoma: candidates for T-cell-based immunotherapies? Cancer Res 58: 4090–4095 9751617

[bib11] Oberthuer A, Hero B, Spitz R, Berthold F, Fischer M (2004) The tumor-associated antigen PRAME is universally expressed in high-stage neuroblastoma and associated with poor outcome. Clin Cancer Res 10: 4307–4313 15240516 10.1158/1078-0432.CCR-03-0813

[bib12] Paydas S, Tanriverdi K, Yavuz S, Disel U, Baslamisli F, Burgut R (2005) PRAME mRNA levels in cases with acute leukemia: clinical importance and future prospects. Am J Hematol 79: 257–261 16044453 10.1002/ajh.20425

[bib13] Radich JP, Dai H, Mao M, Oehler V, Schelter J, Druker B, Sawyers C, Shah N, Stock W, Willman CL, Friend S, Linsley PS (2006) Gene expression changes associated with progression and response in chronic myeloid leukemia. Proc Natl Acad Sci USA 103: 2794–2799 16477019 10.1073/pnas.0510423103 PMC1413797

[bib14] Steinbach D, Hermann J, Viehmann S, Zintl F, Gruhn B (2002a) Clinical implications of PRAME gene expression in childhood acute myeloid leukemia. Cancer Genet Cytogenet 133: 118–123 11943337 10.1016/s0165-4608(01)00570-2

[bib15] Steinbach D, Viehmann S, Zintl F, Gruhn B (2002b) PRAME gene expression in childhood acute lymphoblastic leukemia. Cancer Genet Cytogenet 138: 89–91 12419593 10.1016/s0165-4608(02)00582-4

[bib16] van ‘t Veer LJ, Dai H, van de Vijver MJ, He YD, Hart AA, Mao M, Peterse HL, van der Kooy K, Marton MJ, Witteveen AT, Schreiber GJ, Kerkhoven RM, Roberts C, Linsley PS, Bernards R, Friend SH (2002) Gene expression profiling predicts clinical outcome of breast cancer. Nature 415: 530–536 11823860 10.1038/415530a

[bib17] van de Vijver MJ, He YD, van't Veer LJ, Dai H, Hart AA, Voskuil DW, Schreiber GJ, Peterse JL, Roberts C, Marton MJ, Parrish M, Atsma D, Witteveen A, Glas A, Delahaye L, van der Velde T, Bartelink H, Rodenhuis S, Rutgers ET, Friend SH, Bernards R (2002) A gene-expression signature as a predictor of survival in breast cancer. N Engl J Med 347: 1999–2009 12490681 10.1056/NEJMoa021967

[bib18] Willenbrock K, Kuppers R, Renne C, Brune V, Eckerle S, Weidmann E, Brauninger A, Hansmann ML (2006) Common features and differences in the transcriptome of large cell anaplastic lymphoma and classical Hodgkin's lymphoma. Haematologica 91: 596–604 16670065

